# Nonsuicidal Self-Injury Disorder in Adolescents: Clinical Utility of the Diagnosis Using the Clinical Assessment of Nonsuicidal Self-Injury Disorder Index

**DOI:** 10.3389/fpsyt.2020.00008

**Published:** 2020-02-14

**Authors:** Maria Zetterqvist, Irene Perini, Leah M. Mayo, Per A. Gustafsson

**Affiliations:** ^1^ Center for Social and Affective Neuroscience, Department of Clinical and Experimental Medicine, Linköping University, Linköping, Sweden; ^2^ Department of Child and Adolescent Psychiatry, Region Östergötland, Linköping, Sweden

**Keywords:** nonsuicidal self-injury, disorder, adolescents, Diagnostic and Statistical Manual of Mental Disorders, diagnosis

## Abstract

Nonsuicidal self-injury disorder (NSSID) is a condition in need of further study, especially in adolescent and clinical populations where it is particularly prevalent and studies are limited. Twenty-nine clinical self-injuring adolescents were included in the study. The Clinical Assessment of Nonsuicidal Self-Injury Disorder Index (CANDI) was used to assess prevalence of NSSID. The NSSID diagnosis criteria were met by 62.1% of adolescents. The impairment or distress criterion was least often met. Criteria B and C (assessing reasons for NSSI and cognitions/emotions prior to NSSI) were confirmed by 96–100% of all participants. Adolescents with NSSI in this clinical sample had several comorbidities and high levels of psychopathology. NSSID occurred both in combination with and independently of borderline personality disorder traits as well as suicide plans and attempts. Those with NSSID had a significantly higher cutting frequency than those not meeting full NSSID criteria. Other NSSI characteristics, comorbidity, psychopathology, and trauma experiences did not differ between groups. CANDI was a feasible tool to assess NSSID in adolescents. It is important to use structured measures to assess the validity of the NSSID diagnosis across development in both community and clinical samples. The clinical utility of the NSSID diagnosis is discussed.

## Introduction

Nonsuicidal self-injury (NSSI), i.e., deliberately injuring one's own body tissue without suicidal intent (1), is a significant mental health problem among adolescents. This is especially true in clinical populations, in which 40% or more report NSSI (2). NSSI is also common in non-clinical samples, with prevalence rates estimated to be around 18% (3). There is an ongoing discussion whether NSSI should be considered a separate diagnostic entity (4). Those in favor of a nonsuicidal self-injury disorder (NSSID) diagnosis emphasize the pros of having specified criteria cut-offs. This would enable identification of individuals with more severe NSSI who potentially are at high risk and in need of treatment. Furthermore, a diagnosis would stimulate treatment research and distinguish the condition from borderline personality disorder (BPD) and suicidality. It would also lead to improved communication and conceptual clarity in clinical practice (5, 6).

In 2013, NSSI was included in section III of the fifth version of the Diagnostic and Statistical Manual of Mental Disorders [DSM-5; (7)], as a condition in need of further study, making it a highly relevant research area. As currently proposed, NSSID is a dichotomous diagnosis (8), consisting of six criteria that all have to be met in order for a diagnosis of NSSID to be applicable. The potential diagnosis of NSSID is conceptualized as a condition that can occur with or without other comorbidities, such as BPD, as well as suicidality (9). Preliminary data have shown that NSSID prevalence rates range between 5.6% and 7.6% among non-clinical samples of adolescents (10–12), and 0.2–0.8% in young adults (13, 14). In clinical adolescent self-injuring samples, between 74% and 78% meet full criteria (8, 15).

Since the criteria were published, they have been subject to discussion (e.g., 6, 8, 16–19). Based on recent empirical data, revisions of the criteria have been suggested, including proposals that a dimensional approach to the disorder would be more beneficial (8). Criterion A is a frequency criterion, with NSSI occurring on at least 5 days during the past year. See **Table 1** for full NSSID diagnostic criteria (7). The validity of criterion A has been examined and debated. Recent studies suggest that the cut-off level needs to be increased and/or the time period needs to be decreased to better delimit individuals with more severe NSSI (16–18). Furthermore, severity needs to be taken into account (16–18). Currently, no distinction is made based on severity between five incidents of less serious damage to body tissue by scratching, for instance, and five episodes of severe cutting that need to be stitched. Criteria B and C measure reasons for engaging in NSSI and emotions/cognitions experienced prior to NSSI, respectively. Criterion C further measures preoccupation with and thoughts about NSSI that are difficult to resist that occur before NSSI. Criterion B has shown to be very highly endorsed and does not discriminate sufficiently since it is overinclusive (8, 20). Similarly, criterion C, specifically C1, has shown to be very commonly endorsed, with interpersonal difficulties or negative states preceding the NSSI incident (8). In a study by Washburn and colleagues (8), criterion C1 was significantly associated with several measures of psychopathology and impairment.

**Table 1:** Proposed Nonsuicidal Self-Injury Criteria in DSM-5.

A.	In the last year, the individual has, on 5 or more days, engaged in intentional self-inflicted damage to the surface of his or her body of a sort likely to induce bleeding, bruising, or pain (e.g., cutting, burning, stabbing, hitting, excessive rubbing), with the expectation that the injury will lead to only minor or moderate physical harm (i.e., there is no suicidal intent). Note: The absence of suicidal intent has either been stated by the individual or can be inferred by the individual's repeated engagement in a behavior that the individual knows, or has learned, is not likely to result in death.
B.	The individual engages in the self-injurious behavior with one or more of the following expectations:
1. 2. 3.	To obtain relief from a negative feeling or cognitive state. To resolve an interpersonal difficulty. To induce a positive feeling state.
	Note: The desired relief or response is experienced during or shortly after the self-injury, and the individual may display patterns of behavior suggesting a dependence on repeatedly engaging in it.
C.	The intentional self-injury is associated with at least one of the following:
1. 2. 3.	Interpersonal difficulties or negative feelings or thoughts, such as depression, anxiety, tension, anger, generalized distress, or self-criticism, occurring in the period immediately prior to the self-injurious act. Prior to engaging in the act, a period of preoccupation with the intended behavior that is difficult to control. Thinking about self-injury that occurs frequently, even when it is not acted upon.
D.	The behavior is not socially sanctioned (e.g., body piercing, tattooing, part of a religious or cultural ritual) and is not restricted to picking a scab or nail biting.
E.	The behavior or its consequences cause clinically significant distress or interference in interpersonal, academic, or other important areas of functioning.
F.	The behavior does not occur exclusively during psychotic episodes, delirium, substance intoxication, or substance withdrawal. In individuals with a neurodevelopmental disorder, the behavior is not part of a pattern of repetitive stereotypies. The behavior is not better explained by another mental disorder or medical condition (e.g., psychotic disorder, autism spectrum disorder, intellectual disability, Lesch-Nyhan syndrome, stereotypic movement disorder with self-injury, trichotillomania [hair-pulling disorder], excoriation [skin-picking] disorder).

Reprinted with permission from the Diagnostic and Statistical Manual of Mental Disorders, Fifth Edition, (Copyright ©2013). American Psychiatric Association.

Criterion D ensures that behaviors that are considered socially sanctioned or minor are not included. The diagnostic prerequisite of functional impairment and/or distress caused by the disorder is met by criterion E. The issue of impairment or distress has also been empirically examined (21). Although criterion E is often the least commonly met in several studies (11, 14, 21, 22), it best discriminates those with an NSSID diagnosis from those not meeting full criteria (23). Finally, criterion F describes possible exclusion and differential diagnoses that potentially better explain the self-injurious behavior (7).

The main objective of the diagnosis, i.e., to identify individuals with more severe NSSI, has been examined. Studies that have tried to validate the NSSID diagnosis have shown that it exists both independently of BPD and other disorders (24), as well as comorbidly (8, 15). The criteria can also detect a more severe group compared to individuals with NSSI who do not meet diagnostic criteria. Those with NSSID show greater NSSI versatility, as well as more psychopathology and functional impairment (8, 14, 15, 22, 23, 25). Studies that have examined whether the NSSID criteria could demarcate individuals from those with NSSI in clinical adolescent samples (8, 25) have shown preliminary support for more suicidality in the NSSID group, but also some inconsistencies. The clinical utility of the current diagnostic criteria in clinical samples has also been questioned (8).

Earlier empirical research on NSSID has predominately been done on (young) adults and/or in community/college samples. Only very few studies have focused on clinical and adolescent populations (8, 15, 18, 25). Thus, further research is sorely needed to test the clinical utility of the NSSID diagnosis, as clinical adolescents are those most likely to receive a diagnosis and need treatment planning in clinical practice. The clinical utility of the proposed NSSID criteria is its ability to reliably identify and delimit a group of individuals who are suffering and are impaired by their NSSI. Individuals that have more severe symptoms and risk a negative developmental trajectory are in need of specific treatment.

NSSI characteristics such as methods, frequency and age of onset, clinical psychopathology including comorbid psychiatric diagnoses, BPD, suicidality, emotional awareness and difficulties with emotion regulation, negative life events, and trauma experience are possible variables that are relevant in discriminating between NSSID and NSSI and in examining the clinical utility of the NSSID criteria (26, 27).

Several earlier studies on NSSID have used self-report or other different measures to operationalize the criteria, making comparisons difficult. Clinical interviews developed to assess the NSSID diagnosis have been lacking. The clinical trials preceding DSM-5 resulted in lack of reliability for the NSSID diagnosis (28), which indicates that reliable and valid measures to assess NSSID are crucial to advance the research instead of relying solely on self-report. One such addition is the Clinical Assessment of Nonsuicidal Self-injury Disorder Index (CANDI; 23), which is a semi-structured clinical interview that assesses the full NSSID criteria. CANDI was originally developed for adults and has been psychometrically validated on an adult population (23). The Swedish version has also been used to assess adolescents that participated in an emotion regulation treatment study (29, 30), suggesting that it has potential for this population. There is a need for studies that examine the feasibility of structured measures to assess NSSID, especially in psychiatric adolescent populations, in order to validate and document NSSID prevalence across development, to identify risk and facilitate treatment planning.

To conclude, previous research on the NSSID diagnosis has focused on adult samples, and there are few studies on NSSID in clinical adolescent samples. Structured diagnostic interviews are important for assessment, but these are lacking and little research has been done, especially on psychiatric samples. Advances within this field will help clinicians adequately assess NSSID and avoid over-pathologizing the behavior. Early identification of NSSID may support intervention strategies for those in need and prevent a further negative development of NSSI.

The aim of this explorative pilot study was to investigate the feasibility of assessing DSM-5 NSSID criteria using the structured interview CANDI in a clinical sample of adolescents by examining whether a more severe group could be identified by comparing demographics, NSSI characteristics, psychopathology, and trauma experience of those with an NSSID diagnosis to those with NSSI not meeting full NSSID criteria.

## Method

### Participants and Procedure

Participants with NSSI were recruited from the child and adolescent psychiatric (CAP) clinic in Linköping, Sweden, from June 2016 to March 2018, as part of a study examining neurobiological markers of NSSI in 30 clinical and 30 healthy adolescents which was carried out at the Center for Social and Affective Neuroscience, Linköping University (31). Inclusion criteria for participants were: having engaged in five or more instances of NSSI during the past 6 months, independent of psychiatric diagnosis, and being a female between 15 and 18 years of age. The time period was limited to 6 months to ensure that participants had relatively recent NSSI, since temporal proximity is important when answering questions referring to NSSI characteristics (such as frequency, methods, and functions), in order avoid questionable retrospective recall (32). The lower age limit was set to 15 years to increase chances of obtaining valid responses from measures, some of which were originally developed for adults. We also limited the sample to females since NSSI is more prevalent in females in clinical samples (33). Based on clinical experience we predicted that the sample would include only one to two male participants if males were included. Exclusion criteria were: current or life-time diagnosis of schizophrenia, bipolar or psychotic disorder and/or alcohol/drug dependence, and IQ below 80. Adolescents meeting inclusion criteria were approached with oral and written information about the study. Participants (and parents, if the participant was <18 years) gave written informed consent. The study was approved by the Regional Ethical Board of Linköping (Dnr 2015/273-31; 2016/224-32).

Participants were assessed at the CAP clinic during one or two sessions by the first author, a clinical child psychologist with extensive experience in psychiatric assessment, who evaluated the NSSID diagnosis. Final comorbid psychiatric diagnoses for the clinical sample were made together with the last author, a child psychiatrist, and were based on all available information from diagnostic interviews and medical records, using DSM-5 (7).

Of the 30 adolescents with NSSI that were recruited to the study, one was not assessed with CANDI. Thus, 29 female participants (*M* = 15.9 years, *SD* = 0.77) were included in the present study.

### Psychometric Measures

#### Demographics

Demographic information concerning family status and living conditions, ethnicity and parental education was assessed with questions developed for this study, and from clinical records

#### Nonsuicidal and Suicidal Self-Injury Thoughts and Behaviors

NSSI characteristics (including frequency and means of self-injury) and diagnosis were assessed using the Clinical Assessment of Nonsuicidal Self-injury Disorder Index (CANDI; 23), which is a semi-structured clinical interview that assesses the full NSSID criteria. CANDI includes an initial screen of past year NSSI, both frequency and days. Each diagnostic criterion is assessed using yes/no presence of the criterion and continuous follow-up data are obtained. CANDI was originally developed for adults, and has been psychometrically validated on an adult population demonstrating good interrater reliability and adequate internal consistency (23). The Swedish version has recently been used to assess adolescents who participated in an emotion regulation treatment study (29, 30).

Selected questions from the semi-structured Self-Injurious Thoughts and Behaviors Interview (SITBI; 34) were used to obtain detailed information about suicidal behavior. SITBI assesses the presence, frequency, and characteristics of self-injurious thoughts and behaviors, and has psychometrically shown strong interrater reliability, test–retest reliability, and concurrent validity (34). SITBI has also been validated in Swedish with good psychometric properties (12).

#### Psychopathology

The clinical interview Schedule for Affective Disorders and Schizophrenia for School-Age Children-Present and Lifetime Version (K-SADS-PL; 35) from 2009 was used for DSM-IV diagnoses. K-SADS-PL has three sections: 1) introductory interview; 2) screen interview; 3) eight optional supplements. These refer to: 1) affective disorders; 2) psychotic disorders; 3) anxiety disorders; 4) behavioral disorders; 5) substance use disorders; 6) eating disorders; 7) tic disorders; 8) autism spectrum disorders.

Difficulties in Emotion Regulation Scale (DERS; 36) was used to measure difficulties in identifying and regulating negative emotions and difficulties in using goal-directed behavior while under the influence of negative emotions. The questionnaire consists of 36 items rated on a 5-point Likert-type scale from “almost never” to “almost always.” It has six subscales: difficulties in emotional acceptance, difficulties in goal-directed behavior, difficulties in impulse control, lack of emotional awareness, lack of emotion regulating strategies, and lack of emotional clarity. Higher scores indicate more difficulties with emotion regulation. The questionnaire has been used both clinically and in normative populations for adults and adolescents, and has shown to have high internal consistency, good test–retest reliability, and adequate construct validity. Cronbach's alpha for the clinical adolescents' total score in the present sample was α = 0.78, indicating acceptable internal consistency.

Toronto Alexithymia Scale (TAS-20; 37) was used to assess alexithymia. TAS-20 has 20 items, ranging from totally right to totally wrong on a 5-grade Likert-type scale. The questionnaire comprises three subscales: difficulties identifying emotions, difficulties describing emotions, and difficulties externalizing emotions. Higher scores indicate higher levels of alexithymia. TAS-20 is one of the most commonly used self-report scales for alexithymia and has shown good reliability and validity. In the present clinical adolescent sample, internal consistency for the total TAS-20 was α = 0.70; which is acceptable.

#### Borderline Personality Traits

The Structural Clinical Interview for DSM-IV Personality Disorders (SCID-II; 38) was administered for symptoms of borderline personality disorder (BPD). Both the self-report and interview versions were used in the present study. Cronbach's alpha for the self-report version of BPD was α = 0.85 in the present sample.

#### Traumatic Experiences

The Trauma Symptom Checklist for Children (TSCC; 39) is a self-report questionnaire developed to identify symptoms of traumatic stress in children and adolescents. The questionnaire consists of 54 items and the respondents rate their answers on a 4-point Likert scale from 0 (never) to 3 (almost always). The results are divided into six subscales (depression, anxiety, dissociation, post-traumatic stress, sexual concern, and anger). TSCC has been evaluated on Swedish children and adolescents with good reliability (internal consistency, test–retest) and satisfactory concurrent and criterion-related validity (40). TSCC showed excellent internal consistency for the total scale in the present sample, α = 0.92.

Linköping Youth Life Experience Scale (LYLES; 41) is an instrument for gauging potentially traumatic life events, including adverse childhood circumstances. LYLES contains 23 main questions and includes measures of interpersonal, non-interpersonal, and adverse childhood circumstances. LYLES has been evaluated on Swedish adolescents from the normative population. Its psychometric properties have been shown to be satisfactory (41).

#### Intelligence

An abbreviated version of Wechsler Intelligence Scales, fourth edition for children (42) or adults (43), was used to assess intelligence depending on participants' age.

### Data Analysis

Continuous data were first tested for normality using Shapiro–Wilk. NSSI frequency and reported NSSI reasons and experiences prior to NSSI were not normally distributed. Data were analyzed with descriptive statistics using frequencies, mean values, cross-tabulation with chi-square (χ^2^) and independent samples t-tests. All data were baseline data. For data not meeting requirements for normal distribution, between group comparisons were analyzed with nonparametric Mann–Whitney U test and median was presented. For cross-tabulation of categorical data, Fischer's exact test was used when cells had an expected count less than five. Internal consistency was assessed using Cronbach's alpha (α) for the self-report measures. All statistical analyses were performed using the SPSS 24.0 software package (SPSS Inc, Chicago, IL).

## Results

### Demographic Characteristics

All participating adolescents were female (*n* = 29, 100.0%). Mean age (SD) was 15.9 (0.77) years for the total NSSI group. Adolescents meeting full criteria for the NSSID diagnosis had a significantly higher IQ (*M* = 99.0, *SD* = 8.25) compared to those not meeting full criteria (*M* = 91.0, *SD* = 9.45). There were no significant differences between groups concerning age, family structure, parental education, or ethnicity. Sample characteristics are presented in **Table 2**.

**Table 2:** Participant demographics.

Demographic characteristics	Total NSSI sample *n* = 29 *n* (%)	NSSID *n* = 18 *n* (%)	NSSI *n* = 11 *n* (%)	*p*
Sex				
Female	29 (100.0)	18 (100.0)	11 (100.0)	–
Age				
* m* (*SD*)	15.9 (0.77)	15.8 (0.86)	16.0 (0.63)	0.58ǂ
IQ				
* m* (*SD*)	96.0 (9.42)	99.0 (8.25)	91.0 (9.45)	0.02ǂ
Family structure				1.0†
Married/co-habitant	12 (41.4)	7 (38.9)	5 (45.5)	
Divorced	17 (58.6)	11 (61.1)	6 (54.5)	
Mother's highest level of education	*n* = 28	*n* = 17	*n* = 11	0.41 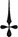
University/college	13 (46.4)	8 (47.1)	5 (45.4)	
Theoretical high school program	2 (7.1)	2 (11.8)	0 (0.0)	
Vocational high school program	12 (42.9)	7 (41.2)	5 (45.4)	
Compulsory school	1 (3.6)	0 (0.0)	1 (9.1)	
Father's highest level of education	*n* = 25	*n* = 16	*n* = 9	0.62 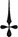
University/college	9 (36.0)	6 (37.5)	3 (3.33)	
Theoretical high school program	2 (8.0)	1 (6.3)	1 (11.1)	
Vocational high school program	11 (44.0)	8 (50.0)	3 (33.3)	
Compulsary school	3 (12.0)	1 (6.3)	2 (22.2)	
Parent born in other country	*n* = 55 4 (7.3)	*n* = 35 4 (11.4)	*n* = 20 0 (0.0)	0.28†

NSSI, nonsuicidal self-injury; NSSID, nonsuicidal self-injury disorder.

ǂ Independent sample t-test. † Fischer’s exact test. 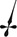 Pearson’s chi-square.

### Self-Injury Characteristics and Comparisons Between NSSI Groups

Of the total sample of adolescents with NSSI, 18 (62.1%) met full criteria for NSSID according to CANDI, while 11 (37.9%) did not. For the proportion of adolescents meeting NSSID criteria see **Table 3**. Criterion A was met by 86.2% of the total sample. Those not meeting criterion A (13.8%), despite the presence of five or more NSSI episodes on different days during the past year, were assessed as having too minor a form of self-injury that did not result in bleeding or bruising. All participants (100.0%) met criteria C, D and F. All but one participant (96.6%) met criterion B, as assessed by CANDI, since she did not confirm any of the suggested or other reasons for engaging in NSSI (**Table 4**). The criterion that was met least often was criterion E, with self-injury being associated with suffering or impairment for 72.4% in this sample. The 21 adolescents (72.4%) that endorsed criterion E mostly acknowledged impairment/distress due to having to cover up scars during summertime or experiencing shame and guilt over cutting, as well as worrying about family members' reactions to their NSSI. For adolescents that did not meet criterion E, distress or impairment was not attributed to NSSI per se, but rather to symptoms associated with other diagnoses or problems.

**Table 3:** Participants meeting NSSID criteria, frequencies and percentages.

Criteria	Total NSSI sample (*n* = 29)	NSSID (*n* = 18)	NSSI (*n* = 11)
Criterion A	25 (86.2)	18 (100.0)	7 (63.6)
Criterion B	28 (96.6)	18 (100.0)	10 (90.9)
Criterion C	29 (100.0)	18 (100.0)	11 (100.0)
Criterion D	29 (100.0)	18 (100.0)	11 (100.0)
Criterion E	21 (72.4)	18 (100.0)	3 (27.3)
Criterion F	29 (100.0)	18 (100.0)	11 (100.0)
NSSID diagnosis	18 (62.1)	18 (100.0)	0 (0.0)

NSSID, nonsuicidal self-injury disorder.

**Table 4:** Self-injury characteristics and comparisons between NSSI groups.

Measure	Total NSSI sample (*n* = 29)	NSSID (*n* = 18)	NSSI (*n* = 11)	*p*
**NSSI**				
Age of onset *m* (*SD*)	13.2 (1.25)	12.9 (1.34)	13.6 (1.03)	0.16ǂ
Number of methods *m* (*SD*)	3.76 (2.13)	4.17 (2.26)	3.09 (1.81)	0.19ǂ
12 months cutting frequency *m* (*SD*), *median*	64.28 (83.36) 30.0	94.94 (93.55) 75.0	14.09 (10.77) 10.0	0.001*
12 months severely scratched frequency *m* (*SD*), *median*	15.07 (29.53) 2.0	22.11 (37.74) 4.0	3.55 (5.92) 0.0	0.16*
12 months prevented wounds from healing frequency *m* (*SD*), *median*	33.72 (79.39) 1.0	51.78 (97.20) 12.5	4.18 (6.19) 0.0	0.13*
12 months punched oneself frequency *m* (*SD*), *median*	15.55 (56.18) 0.0	21.94 (70.87) 0.0	5.09 (10.20) 0.0	0.98*
Three most commonly endorsed reasons for NSSI 0–100% *m* (*SD*), *median*	Decrease negative emotion 80.69 (25.45) 90.0 Punish 70.52 (33.87) 90.0 Distract 51.55 (34.26) 50.0	Decrease negative emotion 87.78 (9.27) 90.0 Punish 72.22 (33.35) 90.0 Distract 48.33 (33.12) 50.0	Decrease negative emotion 69.09 (37.80) 90.0 Punish 67.73 (36.15) 70.0 Distract 56.82 (37.08) 50.0	0.73* 0.62* 0.50*
Three most commonly endorsed emotion/cognition prior to NSSI 0–100% *m* (*SD*), *median*	Sad/depressed 79.93 (23.68) 90.0 Worthless 74.38 (28.51) 90.0 Failure 72.90 (22.40) 80.0	Sad/depressed 80.72 (24.21) 91.50 Worthless 72.06 (27.79) 80.0 Failure 70.22 (25.08) 75.0	Sad/depressed 78.64 (23.88) 90.0 Worthless 78.18 (30.60) 100.0 Failure 77.27 (17.37) 80.0	0.76* 0.28* 0.55*
**Suicidal behaviors**				
Suicide ideation *Frequency (%)*	29 (100.0)	18 (100.0)	11 (100.0)	–
Suicide plans *Frequency (%)*	15 (51.7)	11 (61.1)	4 (36.4)	0.36†
Suicide attempt *Frequency (%)*	10 (34.5)	7 (38.9)	3 (27.3)	0.69†

NSSI, nonsuicidal self-injury; NSSID, nonsuicidal self-injury disorder. ǂ Independent sample t-test (due to small samples analyses were also run with Mann-Whitney U-test with similar results). * Nonparametric Mann-Whitney U-test. † Fischer's exact test.

Mean age of onset for NSSI was 12.9 (*SD* = 1.34) years in the NSSID group (*n =* 18) compared to 13.64 (*SD* = 1.03) years in the NSSI group not meeting full criteria (*n* = 11). Mean number of different NSSI methods used in the total sample was 3.76 (2.13). There were no differences between groups regarding age of onset or number of methods. The only significant difference in self-injury characteristics between groups was the 12-month cutting frequency (*p* = 0.001), where those with NSSID had a significantly higher frequency than those not meeting full NSSID criteria. Other methods of self-injury, such as severely scratching, punching oneself, or preventing wounds from healing, did not differ between groups. See **Table 4**. The three most commonly reported reasons for engaging in NSSI (criterion B) were the same in both groups: to decrease or relieve a negative emotion; to punish yourself; to distract yourself from negative thoughts or feelings. The three most commonly reported emotions and thoughts that were experienced before engaging in NSSI (criterion C) were also the same in both groups: feeling sad, depressed or down; feeling worthless, hopeless, or undeserving; and feeling like a failure or inferior (**Table 4**). There were no differences between groups regarding reported reasons or experiences prior to NSSI. There was considerable co-occurrence of NSSI and suicidality in the present sample. Every adolescent with NSSI (*n* = 29, 100.0%) reported life-time prevalence of suicidal ideation. Suicidal plans were reported by 61.1% of those with NSSID and 36.4% in the NSSI group, but the difference was not statistically significant. Suicide attempts were reported by 34.5% in the NSSID group, and 27.3% of those with NSSI reported having made a suicide attempt. The difference was not significant. See **Table 4**.

The CANDI interview was feasible to administer to this clinical sample of adolescents. The questions were understood by the participants. It was possible to make a final assessment of NSSID based on the information gathered in the CANDI interview. Several of the self-injury methods listed in the CANDI, however, such as using acid, bleach or breaking bones, were not acknowledged by any of the adolescent participants in this sample.

### Psychopathology, Traumatic Experiences, and Comparisons Between NSSI Groups

Of the total sample (*n* = 29), 51.7% met criteria for attention deficit hyperactivity disorder, hyperactive/impulsive or inattentive (ADHD/ADD) subtype, and 48.3% met criteria for depression. Comorbid anxiety disorders (44.8%) were also common, as were borderline personality disorder (BPD) traits (41.4%). The mean total number of comorbid psychiatric diagnoses was 2.31 (*SD* = 1.00). NSSID was comorbid with several diagnoses, such as depression (*n* = 7, 38.9%), anxiety disorder (*n* = 7, 38.9%), borderline traits (*n* = 8, 44.4%), and ADHD/ADD (*n* = 10, 55.6%). NSSID also occurred independently of BPD in 10 cases (55.6%). Adolescents in the NSSI group who did not meet full NSSID criteria also had several comorbidities: depression (*n* = 7, 63.6%), anxiety disorder (*n =* 6, 54.5%), borderline traits (*n* = 4, 36.4%), and ADHD/ADD (*n* = 5, 45.5%). There were no significant differences between comorbid diagnoses in the NSSID and the NSSI group. The psychiatric diagnoses for the total sample and respective group (NSSID and NSSI) are presented in **Table 5**.

**Table 5:** Psychopathology, traumatic experiences, and comparisons between NSSI groups.

Psychiatric diagnoses*	Total NSSI sample (*n* = 29) *n* (%)	NSSID (*n* = 18) *n* (%)	NSSI (*n* = 11) *n* (%)	*p*
Depression	14 (48.3)	7 (38.9)	7 (63.6)	0.36 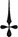
Anxiety disorder	13 (44.8)	7 (38.9)	6 (54.5)	0.47†
Posttraumatic stress disorder	1 (3.4)	1 (5.6)	0 (0.0)	1.00†
Borderline traits	12 (41.4)	8 (44.4)	4 (36.4)	0.72†
Eating disorder	6 (20.7)	3 (16.7)	3 (27.3)	0.65†
ADHD/ADD	15 (51.7)	10 (55.6)	5 (45.5)	0.89 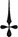
High-functioning autism	3 (10.3)	3 (16.7)	0 (0.0)	0.27†
ODD/CD	3 (10.3)	3 (16.7)	0 (0.0)	0.27†
Ever had inpatient psychiatric care	6 (20.7)	6 (33.3)	0 (0.0)	0.06†
Mean total diagnoses *m* (*SD*)	2.31 (1.00)	2.33 (1.08)	2.27 (0.90)	0.98ǂ
SCID-II self-report *m* (*SD*)	5.93 (2.83)	5.44 (2.98)	6.73 (2.49)	0.20ǂ
SCID-II interview *m* (*SD*)	3.14 (2.56)	2.78 (2.71)	3.73 (2.28)	0.29ǂ
DERS *m* (*SD*)	123.96 (23.25)	123.0 (24.76)	125.89 (21.15)	0.88ǂ
TAS-20 *m* (*SD*)	63.22 (11.35)	63.56 (11.62)	62.56 (11.46)	0.96ǂ
TSCC *m* (*SD*)	62.63 (20.70)	59.56 (19.46)	68.78 (22.88)	0.35ǂ
LYLES *m* (*SD*)	8.33 (3.63)	7.78 (3.80)	9.44 (3.17)	0.44ǂ

NSSID nonsuicidal self-injury disorder; NSSI, nonsuicidal self-injury; CDRS-R, Children's Depressive Rating Scale–Revised; SCID-II, Structural Clinical Interview for DSM-IV Personality Disorders; LYLES, Linköping Youth Life Event Scale; TSCC, Trauma Symptom Checklist for Children; TAS-20, Toronto Alexithymia Scale; DERS, Difficulties in Emotion Regulation Scale; ADHD/ADD, attention deficit hyperactivity disorder/attention deficit disorder; ODD, oppositional defiant disorder; CD, conduct disorder. * Every patient could have more than one diagnosis. 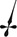 Chi-square with Yates correction. † Fischer's exact test. ǂ Independent samples t-test (due to small samples analyses were also run with Mann-Whitney U-test with similar results).

Six of the 18 adolescents (33.3%) in the NSSID group and none (0.0%) in the NSSI group had been in inpatient care. This was not a significant difference, but a trend (*p* = 0.06). See **Table 5**. There were no significant differences between groups regarding psychopathology, such as borderline symptom scores, self-report or interview assessment, emotion regulation difficulties, alexithymia, or clinician-rated depressive symptoms.

Concerning traumatic experiences, the total number of self-reported negative life events or traumatic symptoms did not differ significantly between the groups (**Table 5**).

## Discussion

The clinical utility of the NSSID diagnosis was examined, and the feasibility of using semi-structured CANDI to assess diagnosis criteria in a clinical sample of adolescents with NSSI. Although originally developed for adults, CANDI was a helpful measure in assessing NSSID and NSSI characteristics in this clinical adolescent sample, and thus well suited for this purpose. Of the clinical adolescents with NSSI in this sample, 62.1% met full criteria for NSSID, with criterion E being least commonly met. Cutting frequency significantly discriminated those with NSSID from those with NSSI not meeting full criteria. Those with NSSID did not differ from those with NSSI not meeting full criteria on other self-injury characteristics or psychopathology. NSSID occurred both independently and together with other psychiatric disorders and suicidality, as did NSSI.

### Self-Injury Characteristics

The NSSID prevalence rate of 62.1% in this clinical sample of self-injuring adolescents is slightly lower than earlier studies of NSSID in clinical self-injuring adolescents, which have found NSSID rates of 74–78% (8, 14). The present sample was predominately an outpatient sample, while the studies from both Glenn and Klonsky and Washburn and colleagues had more severe inpatient participants who were in treatment specifically for NSSI, which is a possible explanation for the higher prevalence. Other prevalence studies of NSSID in adults have shown result rates ranging between 37 and 46.2% in self-injuring clinical college samples and psychiatric outpatient samples, respectively (22, 23). The prevalence rates in the current study thus fall somewhere in between, which is tentatively plausible and contributes further empirical adolescent data toward the NSSID diagnosis.

Those not meeting criterion A (13.8%), despite the presence of five or more NSSI episodes on different days during the past year, were assessed as having too minor a form of self-injury that did not result in bleeding or bruising. CANDI thus identified individuals who did not meet criterion A but had engaged in NSSI during the past 6 months.

Criteria B and C were met by nearly every subject (96.6% and 100%), which, together with similar earlier findings (20), questions the clinical utility of these criteria. The present study also confirmed previous studies (22, 25) concerning criterion E, which had the lowest endorsement (72.4%). This has also shown to be the case in community samples of adolescents (11, 12). Criterion E is a necessary prerequisite for discriminating those meeting NSSID criteria from those who do not (21, 23), since the diagnosis needs to identify those who are impaired by their symptoms. The impairment/distress criterion is thus central to any diagnosis. The clinical utility of this criterion needs to be examined further, however, as many adolescents tend to see their NSSI as helpful rather than distressing (21). In this study the participants tended to attribute their impairment or distress to their comorbid conditions rather than to NSSI per se.

The only significant difference between those with NSSID and those with NSSI was for cutting frequency. Some of the few earlier studies that have investigated the NSSID diagnosis have identified a more serious group concerning NSSI characteristics and psychopathology (23) using the NSSID criteria, but there have also been some inconsistencies concerning the validity and limited clinical utility in clinical samples as the diagnosis is currently formulated (8, 18). When data in the current study were examined there was a higher proportion of adolescents with suicidal plans in the NSSID group compared to those without an NSSID diagnosis, which tentatively supports the presence of a somewhat more severe group among those who met all NSSID criteria. Lack of statistical significance is probably due to the small sample size, so interpretations need to be made with caution.

### Psychopathology and Traumatic Experiences

Results from this study confirm earlier research, where NSSID in clinical samples has been shown to exist both together with and independently of different psychiatric conditions and suicidality (8, 15, 24). This is an important delineation when conceptualizing NSSID, and strengthens the validity of the diagnosis. The most common comorbid conditions in the total sample were ADHD/ADD and depression. Psychopathology did not delimit those with NSSID from those with NSSI not meeting full criteria: depression (38.9% vs 63.6%), ADHD (55.6% vs 45.5%), anxiety disorders (38.9% vs 54.5%), and borderline traits (44.4% vs 36.4%), respectively. Similarly, In-Albon and colleagues did not find diagnostic differences between NSSID and a group with NSSID not meeting full criteria (25). An earlier study that used CANDI in a community sample of young adults with recurrent NSSI (23) did however find some diagnostic differences between the two groups. Those with NSSID had more BPD, bipolar disorder, PTSD, social anxiety disorder, and alcohol dependence compared to those not meeting full criteria. Some diagnostic differences were also found by Kiekens et al. (14), who compared NSSID with a sub-threshold NSSID group. These differences between studies might be due to different samples (adolescents compared to young adults; psychiatric compared to community sample with recurrent NSSI).

In the present sample there were no differences between the NSSID group and the NSSI group on measures of alexithymia or difficulties with emotion regulation. Both groups reported high scores on these measures. In an earlier study by Lüdtke et al. (27) on an inpatient adolescent sample, alexithymia was a significant predictor of NSSID. Difficulties with identifying and describing feelings could potentially increase endorsement of criteria B and C in the NSSID diagnosis.

There was also a trend for those with NSSID (33.3%) in this study to have more experience of inpatient care than those with NSSI (0.0%), which is an indicator of severity.

Traumatic symptoms, as assessed with TSCC, were high in the present total sample (*M* = 62.63, *SD* = 20.70) compared to a previous study of a clinical (*M* = 52.6, *SD* = 23.9) and normative group (*M* = 30.4, *SD* = 18.7) of Swedish adolescents (40). Trauma experience did not separate those with NSSID from those with NSSI, nor did negative life events. This is in line with the earlier study by Lüdtke and colleagues (27), who found that childhood maltreatment and dissociation did not predict a diagnosis of NSSID.

A potential interpretation of the results in the present study could be that the NSSID diagnosis is less useful in discriminating between groups in self-injuring clinical samples with several comorbidities and a high level of psychopathology, compared to community/college samples or clinical samples which includes participants who do not self-injure. This interpretation is similar to the findings of Washburn and colleagues (8). Kiekens and colleagues (14) recently examined NSSID in a large college sample and found a dose-response relationship between NSSI recency and severity and mental disorders and suicidality, where the strength of the association was smaller in the sub-threshold NSSID group compared to those meeting full NSSID criteria.

One assumption could be that since all the individuals in clinical samples are in a severe condition, often with trauma experiences, comorbidity, suicidality, and functional impairment, NSSID as currently defined will not discriminate as clearly within clinical relative to community samples. Thus the clinical utility of the NSSID diagnosis is more limited in identifying a group in need of intervention in this context. Notwithstanding, diagnostic criteria and cut-offs need to be the same regardless of type of sample. If cut-offs were based on empirically derived thresholds in clinical samples, the cutoffs would be higher and would then identify a much more severe group. In that case, there would potentially be a risk of missing NSSID sub-threshold individuals who are also struggling with NSSI and require treatment to avoid an escalating negative trajectory. The NSSID diagnosis would, however, perhaps benefit from different cut-offs based on severity ratings, similar to depression, for example, with minor, moderate and severe conditions. This could potentially increase utility in clinical samples. One such attempt was made by Muehlenkamp et al. (18), who investigated differences between groups with low (1–4), moderate (5–24) and high (≥25) NSSI frequency.

This study contributes important information on the use of a structured clinical measure to assess prevalence rates of NSSID in clinical adolescents, in addition to self-report measures of the criteria in community samples. Although this study focused on a previously somewhat neglected sample in the research area of NSSID, the study has some limitations that need to be addressed. First, the sample size was small with potential type II errors in the statistical subgroup analyses. Also, the multiple comparisons could lead to type I errors. Secondly, the cross-sectional design prevents longitudinal or causal conclusions, such as predicting risk on the basis of the NSSID diagnosis. Thirdly, no validated cut-off values for CANDI were reported, hence the interpretation of the clinical utility of the CANDI is limited. Fourthly, since only girls were included in the study, the findings are restricted in generalizability to other samples. Fifthly, the age group was homogeneous and therefore the feasibility and clinical utility of CANDI for a younger age group cannot be assessed. Sixthly, no other measure of NSSI was used and thus concurrent validity could not be examined. The diagnosis of NSSID was made by one person and inter-rater reliability is therefore lacking.

The NSSID diagnosis is a potential facilitator in identifying risk and planning treatment. More studies investigating the validity of the criteria in larger clinical samples of adolescents are needed. Furthermore, the clinical utility of the diagnosis, and the potential to discriminate between those meeting full criteria NSSID disorder and those who do not, need to be examined further in order not to over-pathologize the behavior. A prospective area for research could be predictively examining whether those who are identified with NSSID are at higher risk of future self-injury, general psychopathology or a high consumption of health care. Results in this field would contribute important data toward establishing clinical utility.

To conclude, CANDI was a feasible tool to evaluate NSSI as an independent disorder in this psychiatric outpatient sample of adolescents. The NSSID diagnosis discriminated between those with and without NSSID in this NSSI sample by cutting frequency, and possibly by inpatient care and suicidal plans, where those with NSSID were a more severe group. However, patients with NSSI (sub-threshold) not meeting full NSSID criteria showed similar levels of psychopathology and NSSI characteristics as patients with NSSID in this clinical sample.

## Data Availability Statement

The datasets generated for this study will not be made publicly available. We do not have participants' consent to share raw data publicly. The data that has been used is confidential and individual participants' could potentially be identified if the dataset is shared.

## Ethics Statement

The study involved human participants and was reviewed and approved by the Regional Ethical Board of Linköping (Dnr 2015/273-31; 2016/224-32). Written informed consent to participate in this study was provided by the participants and the participants’ legal guardian/next of kin.

## Author Contributions

MZ, IP, LM, and PG designed research. MZ performed research. MZ analyzed data and made tables. MZ, IP, LM, and PG wrote the paper.

## Funding

This research was supported by The Swedish Research Council (538-2013-7434) and the ALF Grants, Östergötland County (LIO-535931; LIO-520131).

## Conflict of Interest

The authors declare that the research was conducted in the absence of any commercial or financial relationships that could be construed as a potential conflict of interest.
